# Supplementary material for: Molecular and Functional Analysis of U-box E3 Ubiquitin Ligase Gene Family in Rice (Oryza sativa)
Source: Int J Mol Sci. 2021 Nov 8;22(21):12088. doi: 10.3390/ijms222112088 (PMC8584879; doi:10.3390/ijms222112088)

Supplementary Figure S1. Validation of RNA-sequencing data by quantitative real-time PCR (qRT-PCR). Correlation of data between RNA-seq and qRT-PCR techniques on abiotic stress treatment (A) and biotic stress treatment (B).

A)

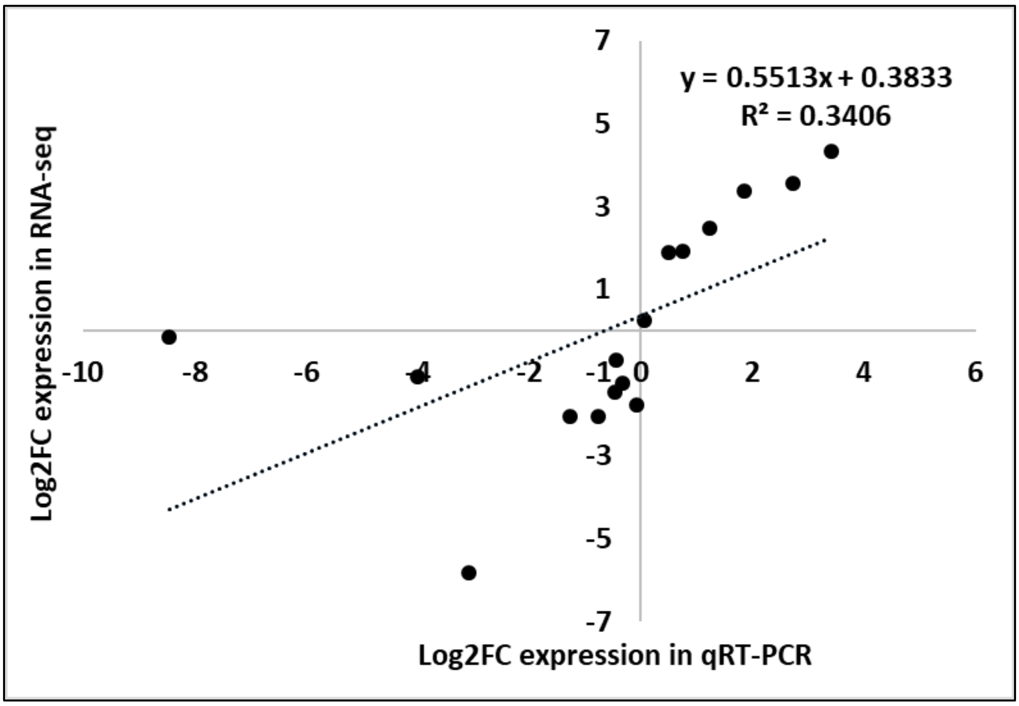

B)

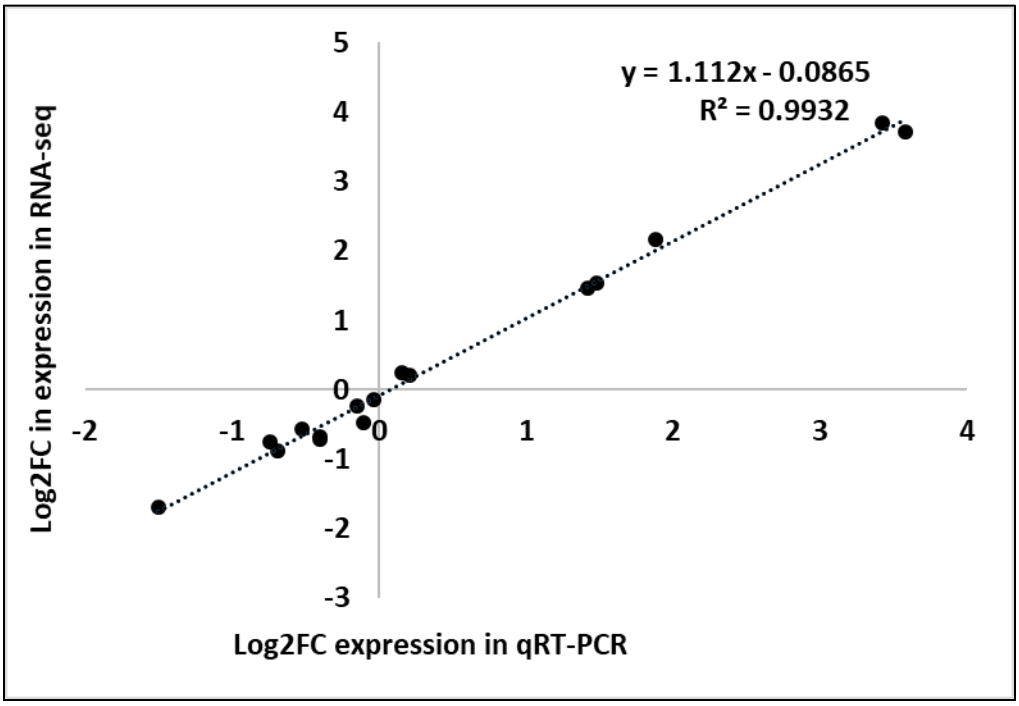

Supplement: Supplementary file 1 [file ijms-22-12088-s001.zip › Supplementary Figure S1.pdf]
